# Th1-Dependent *Cryptococcus*-Associated Immune Reconstitution Inflammatory Syndrome Model With Brain Damage

**DOI:** 10.3389/fimmu.2020.529219

**Published:** 2020-09-29

**Authors:** Yee Ming Khaw, Nupur Aggarwal, William E. Barclay, Eunjoo Kang, Makoto Inoue, Mari L. Shinohara

**Affiliations:** ^1^Department of Comparative Biosciences, University of Illinois at Urbana-Champaign, Urbana, IL, United States; ^2^Neuroscience Program, University of Illinois at Urbana-Champaign, Urbana, IL, United States; ^3^Department of Immunology, Duke University School of Medicine, Durham, NC, United States; ^4^Department of Molecular Genetics and Microbiology, Duke University School of Medicine, Durham, NC, United States

**Keywords:** immune reconstitution inflammatory syndrome, *Cryptococcus neoformans*, interferon-γ, aquaporin-4, Th1 cells, astrocytes, neuroinflammation

## Abstract

*Cryptococcus*-associated immune reconstitution inflammatory syndrome (C-IRIS) is identified upon immune reconstitution in immunocompromised patients, who have previously contracted an infection of *Cryptococcus neoformans* (*Cn*). C-IRIS can be lethal but how the immune system triggers life-threatening outcomes in patients is still poorly understood. Here, we establish a mouse model for C-IRIS with *Cn* serotype A strain H99, which is highly virulent and the most intensively studied. C-IRIS in mice is induced by the adoptive transfer of CD4^+^ T cells in immunocompromised *Rag1*-deficient mice infected with a low inoculum of *Cn.* The mice with C-IRIS exhibit symptoms which mimic clinical presentations of C-IRIS. This C-IRIS model is Th1-dependent and shows host mortality. This model is characterized with minimal lung injury, but infiltration of Th1 cells in the brain. C-IRIS mice also exhibited brain swelling with resemblance to edema and upregulation of aquaporin-4, a critical protein that regulates water flux in the brain in a Th1-dependent fashion. Our C-IRIS model may be used to advance our understanding of the paradoxical inflammatory phenomenon of C-IRIS in the context of neuroinflammation.

## Introduction

Immune reconstitution inflammatory syndrome (IRIS) is a pathological condition whereby a recovering immune system paradoxically worsens the patient’s condition by responding excessively to a previously acquired infection (1, 2). IRIS has been reported in patients, who are recovering from an immunocompromised condition and pre-infected with fungi such as *Cryptococcus, Candida, Aspergillus*, as well as mycobacteria and viruses (3–5). Among these, *Cryptococcus-*associated IRIS (hereinafter “C-IRIS”) is one of the most prevalent IRIS subtypes, and is reported to cause rapid wasting and mortality in immunocompromised patients after immune reconstitution (4, 6–8). Approximately 25% of HIV-infected patients who underwent antiretroviral therapy (6, 9, 10) and ∼5% of immunocompromised patients who had received a solid-organ transplant develop C-IRIS (11). A meta-analysis showed that while lethality was 4.5% for all types of IRIS conditions, lethality for C-IRIS was shown to be 20.8% (8). Not only in AIDS patients, C-IRIS is also reported in some multiple sclerosis (MS) patients. These MS patients had *Cryptococcus* infection prior to discontinuing Natalizumab treatment (12). Natalizumab is an integrin α4 antibody, which prevents T cell migration into the central nervous system (CNS). Therefore, cessation of Natalizumab treatment is considered to allow T cell influx to the CNS. These observations suggest that the history of *Cryptococcus* infection before the influx of CD4^+^ T cells to the CNS may be a crucial feature of C-IRIS induction. Interestingly, C-IRIS also manifests in postpartum women (13, 14). During pregnancy, increased estrogen suppresses the immune system (15) and makes the host vulnerable to fungal infections (16). In the postpartum phase, the immune system is reactivated upon normalization of the estrogen levels, thus triggering C-IRIS development (17). Collectively, these reports suggest that clinical conditions typified by a rapid immune recovery are conducive to the development of C-IRIS.

The CD4^+^ T cell is a key player in driving C-IRIS disease development (18, 19). Patients with C-IRIS typically present with a high number of circulating CD4^+^ T cells, particularly of the Th1 subtype, as well as upregulation of pro-inflammatory cytokine production coupled with clinical pathologies of pulmonary dysfunction, CNS lesions, and brain edema (6–8, 20). Exuberant secretion of cytokines, or a ‘cytokine storm,’ is proposed to underlie C-IRIS (21). However, cytokine levels in the cerebrospinal fluid at the time of C-IRIS is similar to those during initial *Cryptococcus* meningitis among non-C-IRIS patients (7, 22), suggesting an alternative mechanism, rather than cytokine storm, to explain the CNS symptoms of C-IRIS.

Currently, NSAIDs (nonsteroidal anti-inflammatory drugs) and corticosteroids are prescribed to suppress excessive inflammation in C-IRIS patients (5), but such immunosuppressive treatments may impair a response to the existing infection and increase susceptibility to new infections (23, 24). The lack of effective treatments for C-IRIS stems, at least in part, from poor understanding of C-IRIS pathogenesis. To understand the C-IRIS pathogenesis, an animal model which accurately represents the human condition of C-IRIS is essential.

To that end, we report a mouse model for C-IRIS using *Cn* serotype A, which is widespread in the environment (25) and the most commonly identified in HIV patients (26–28). This mouse model presents phenotypes similar to human C-IRIS symptoms, such as systemic upregulation of some inflammatory cytokines and brain edema. Our finding addresses the urgent need for a model to investigate specific cellular and molecular mechanisms underlying C-IRIS.

## Materials and Methods

### Mice

*Rag1*^–/–^ (JAX 002216) and *Ifng*^–/–^ (JAX 008228) mice of the C57BL/6J background and C57BL/6J (JAX 000664) mice were purchased from The Jackson Laboratory. Mice were kept under specific pathogen-free conditions with a 12-h light/dark cycle. Sterile food and water were given *ad libitum* in concordance with the recommendations for the health monitoring of mice. Healthy male and female mice aged 16–20 weeks were randomly selected for use in this study. No sex-specific outcomes were observed. The study was performed under approvals of the Duke University Institutional Animal Care and Use Committee (protocol number A088-18-04) and University of Illinois Institutional Animal Care and Use Committee (protocol number 19171).

### C-IRIS Induction

*Cn*H99 was cultured on YPD (yeast extract 1%, peptone 2%, and dextrose 2%) plates or YPD liquid medium at 30°C with 150 rpm shaking overnight (29). Protocol optimization brought the following methods to be the most reproducible and clinically relevant: *Rag1*^–^*^/^*^–^ mice of 16–20 weeks old were intranasally infected with *Cn*H99 (100 yeasts in 30 μl PBS). CD4^+^ T cells were intravenously transferred (10^6^ cells in 200 μl PBS with 2% FBS) into *Cn*H99 pre-infected mice 3 weeks after *Cn*H99 infection, unless otherwise noted. The C-IRIS experiment scheme with three control groups (naïve, infection alone, T cell transfer alone) is presented in the Supplementary Figure 1A. CD4^+^ T cells were isolated from the spleen and inguinal/axillary lymph nodes of naïve C57BL6 mice (6–8 weeks old) and enriched by negative selection using microbeads (STEMCELL, CD4^+^ cells enrichment). The purity of isolated CD4^+^ T cells was 90–95%. Enriched CD4^+^ T cells included 70.5% naïve (CD62L^+^CD44^–^), 11.3% central memory (CD62L^+^CD44^*hi*^), 13.2% effector (CD62L^–^CD44^*hi*^) CD4^+^ T cells (*n* = 3 average), and 9.1% of Treg cells (CD3^+^CD4^+^Foxp3^+^). Mice were monitored daily, and body weights were recorded.

### ELISA for Serum and Brain Cytokine Levels

Serum and brain cytokine concentrations (IL-6, IL-1β, TNFα, and/or IFNγ) were evaluated using ELISA kits (R&D; DY406 for IL-6, DY401 for IL-1β, DY410 for TNFα, DY823405 for IFNγ). For brain cytokine analysis, the hindbrain region (Bregma −5.0 to −8.0 mm) was dissected with a clean blade and added ice-cold 300 μl RIPA buffer (Fisher Scientific, PI89901) with Halt^TM^ Protease Inhibitor Cocktail (Fisher Scientific, PI78425). Tissues were homogenized and centrifuged for 20 min at 13,000 rpm at 4°C. Protein concentrations were evaluated by the BCA protein quantitation (Fisher Scientific, 23227). All ELISA assays were performed as suggested by manufactures’ instructions.

### Evaluation of Fungal Burdens

Mice were euthanized by CO_2_ inhalation, and lung and brain were removed. For the enumeration of fungal load, organs were homogenized in 1 mL PBS. Serial dilutions of the homogenates were plated on YPD plates and incubated for 48 h at 30°C to evaluate colony-forming units (CFU).

### Leukocytes Isolation From the Brain and Lung

To prepare cells from the lung and brain, tissues were excised and minced in PBS supplemented with collagenase D (1 mg/ml). Minced tissues were incubated for 30 min at 37°C, filtered through 80-μm mesh, then centrifuged at 4°C. We performed hemolysis for cells obtained from the lung. To isolate mononuclear cells from brain and lungs, cells were resuspended in 30% Percoll (in PBS) laid over 70% Percoll, and centrifuged for 20 min at room temperature.

### Cell Staining for Flow Cytometry and Analyses

Before cell staining with antibodies, Fc receptors were blocked with Fc Block (BD Pharmingen) for 7 min on ice. Cells were stained with fluorochrome-conjugated specific antibodies for 30 min on ice, washed, fixed with 4% paraformaldehyde, and analyzed by flow cytometry within 24 h. For intracellular cytokine staining, cells were treated with PMA (10 ng/ml) and Ionomycin (1 μg/ml) for 4 h, including the BD GolgiPlug^TM^ Protein Transport Inhibitor (BD Biosciences BD 555029) treatment in the last 2 h. Cell surface markers were stained first, then intracellular cytokine staining was performed with Cytofix/Cytoperm kit (BD Biosciences; to stain IFN-γ and IL-17) and FOXP3 Fix/Perm Kit (BioLegend; to stain Foxp3). LIVE/DEAD^TM^ fixable dead cell stain kit (Invitrogen) was used to gate out dead cells. Antibodies for flow cytometry are listed in Supplementary Table 1. Flow cytometry data were collected with Cytek Aurora or FACS Canto II (BD) and analyzed with the FlowJo software (Treestar) or FCS Express (De Novo). In lung-derived leukocytes, DC or interstitial or exudate macrophages (DC/iMΦ/eMΦ) were identified as CD45^+^CD11c^+^F4/80^–^, alveolar macrophages (AM) as CD45^+^CD11c^+^F4/80^+^, inflammatory macrophage/monocyte as CD45^+^Ly6C^+^CD11b^+^, neutrophils as CD45^+^Ly6G^*hi*^CD11b^+^, and CD4^+^ T cells as CD45^+^CD3^+^CD4^+^. Gating strategy for lung myeloid cells in the lung is provided in Supplementary Figure 1C. In brain-derived leukocytes, CD4^+^ T cells were identified as CD45^+^CD3^+^CD4^+^, microglia as CD45^*lo*^F4/80^+^, macrophage as CD45^*hi*^F4/80^+^, and neutrophils as CD45^+^Ly6G^+^. Gating strategy to separate microglia from infiltrated macrophages/monocytes is shown in Supplementary Figure 3A. Representative flow cytometry results detecting intracellular IFNγ, IL-17, and Foxp3 in brain-infiltrated CD4^+^ T cells are found in Supplementary Figure 3B.

### Histology of the Lung and Brain, and Quantitative Evaluation of Images

Organs were harvested after perfusion with 4% paraformaldehyde in PBS and embedded into paraffin blocks. Tissue slices (10 μm) were stained with 0.1% Mayer’s Hematoxylin (H&E) for 20 min, rinsed in cool running ddH_2_O for 5 min, then stained with Eosin (0.5% in 95% EtOH) for 12 s. H&E images were acquired using a brightfield microscope with 4× objective. “Area of distal airspaces as percentage (%) of total distal lung area” was evaluated as described in a previous study (30) using ImageJ by manually outlining the areas using a wand tool. Brain vacuolization areas were analyzed with a similar quantitative approach. One data point/mouse in quantitation of histological images is an average value of at least 16 fields (>2 fields/section, 8 sections/mouse) for the lung and at least 3 fields (>3 sections/mouse) for the brain. At least 3 mice/group were used for lung H&E. At least 5 mice/group were used for brain H&E.

### Quantitative Evaluation of Brain Edema

To evaluate brain edema (swelling), circumferences of midbrain (around bregma −1.9 mm) and hindbrain (around bregma −6.3 mm) were measured with manufactured silk and ruler with 0.1 mm accuracy. To minimize variation in measurement, a single person performed the process, and used average values from repeated measurement per sample.

### AQP4 Immunohistochemistry

Brains were perfused, fixed overnight in 4% paraformaldehyde, and cryoprotected by a 30% sucrose treatment in ddH_2_O, before being frozen in the OCT compound (Sakura, Japan). Coronal tissue slices (30 μm) were stained with AQP4 antibody (Novus, 1:500) and goat anti-rabbit Alexa-488 secondary antibody (1:500). Product information of antibodies is listed in Supplementary Table 1. Image fields of superior part of the brainstem (bregma −5.80 mm – bregma −6.24) were acquired by a fluorescent microscope (Leica DM 2000, IL, United States). AQP4 expression was quantified as the area stained with AQP4 antibody over total area with the ImageJ software by converting images to gray scale and being analyzed with the “thresholding” approach^1^. All were blinded during the assessment, and at least four sections of brain stem of each animal (*n = 3–5*/group) were evaluated by one individual.

### Astrocyte Cell Line Culture

Mouse astrocyte cell line C8-D30 (ATCC, CRL-2534; 10^6^ cell/well) was seeded in 24-well plates. One day after seeding, rIFNγ, rIL-17, rIL-6, rIL-1β (10 ng/ml each), or *Cn*H99 (10^7^ yeasts/well) were added to cell culture, and cells were incubated for 5 h at 37°C. Cells were used for RT-qPCR.

### RNA and cDNA Preparation for Standard qPCR Analyses

Total RNA was extracted from cells with RNeasy Kit (Qiagen). cDNA synthesis was performed with qScript cDNA SuperMix (Quanta). qPCR analysis was performed with KAPA-SYBR-FAST (KAPA BioSystems) with an initial denaturing step at 95°C for 3 min, followed by 35 cycles of a denaturation step (94°C for 3 s) and an annealing/extension step (60°C for 30 s). The relative amounts of qPCR products were determined with the ΔΔ*Ct* method with *Actb* (β-actin) as an internal control. Primers are listed in Supplementary Table 2.

### Statistical Evaluations

Sample sizes, numbers of animals, and factors used for statistical evaluations are indicated in figure legends and this Methods section. Student’s *t*-test (two-tailed, if not indicated otherwise) was performed for two group comparison. In case of comparisons of multiple groups, one-way ANOVA and the Tukey *post-hoc* test were performed. Log-rank analysis were used to statistically evaluate survival curve data. *P*-values of 0.05 or less were considered statistically significant. Statistical analyses and graphical presentations were computed with GraphPad Prism software (GraphPad, La Jolla, United States).

## Results

### Experimental C-IRIS Condition Results in Clinical Manifestations of Weight Loss and Death

**FIGURE 1:**
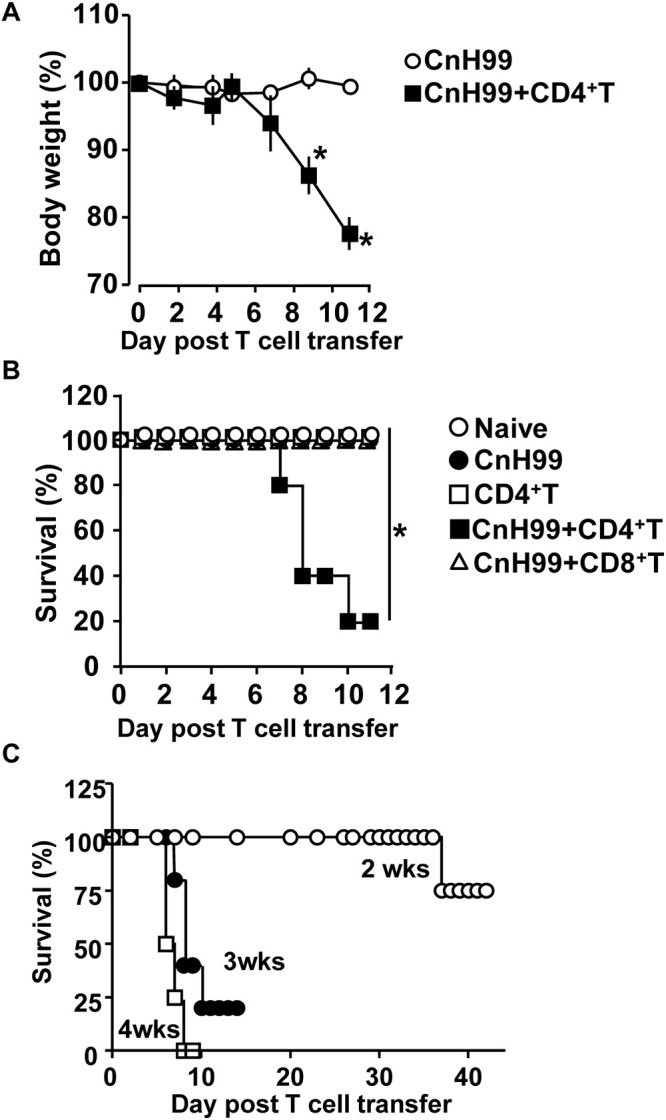
Weight loss and mortality with C-IRIS. **(A)** Comparison of mouse body weights between groups that had infection alone (CnH99) vs. C-IRIS (CnH99 + CD4^+^ T). CnH99 (100 yeasts/mouse) was intranasally infected 3 weeks before CD4^+^ T cell transfer. **(B)** Mouse survival comparing five groups; 4 groups with the protocol indicated in Supplementary Figure 1A, and another group with CnH99 infection and CD8^+^ T cell transfer. Log-rank analysis were used for statistical evaluation. **(C)** C-IRIS mouse survival to evaluate the *Cn*H99 infection period before CD4^+^ T cell transfer. Infection periods tested are 2 weeks (○), 3 weeks (•), and 4 weeks (◻). *n = 5–10* mice per group for all experiments. Results are representatives of at least two independent experiments; *p < 0.05.

*Cryptococcus*-associated immune reconstitution inflammatory syndrome is a complex disorder with high mortality risk in a significant proportion of immunocompromised persons with a history of cryptococcosis (6). To mimic clinical symptoms of C-IRIS, *Rag1*^–/–^ mice were reconstituted with CD4^+^ T cells (10^6^ cells/mouse) by adoptive transfer 3 weeks after infection with *Cryptococcus neoformans* serotype A H99 (CnH99, 100 yeasts/mouse) (Supplementary Figure 1A), unless otherwise noted. While control mice (CnH99-infection alone) did not lose weights between week 3 and 4, 80% of C-IRIS mice (CnH99 + CD4^+^ T) approached the humane endpoint (∼30% weight loss) within 12 days after T cell transfer (Figure 1A). Only 20% of C-IRIS mice survived beyond 10 days after T cell transfer. In contrast, all control mice survived (naïve, CnH99-infection alone, and CD4^+^ T cell transfer alone) in the experiment period (Figure 1B). Interestingly, adoptive transfer of CD8^+^ T cells (10^6^ cells/mouse) did not induce mortality (Figure 1B), emphasizing that the pathogenic role is specific to CD4^+^ T cells in C-IRIS. Next, we examined if the period of *Cn* infection before T cell transfer affects C-IRIS severity. Mice pre-infected with CnH99 for 2 weeks before CD4^+^ T cell transfer had significantly greater survival compared to mice with pre-infection period of 3 or 4 weeks (Figure 1C). These results indicate that C-IRIS mortality is induced specifically by CD4^+^ T cells and that the C-IRIS becomes more severe with extended periods of *Cn* infection.

### C-IRIS Heightens Serum Levels of Some Inflammatory Cytokines

**FIGURE 2:**
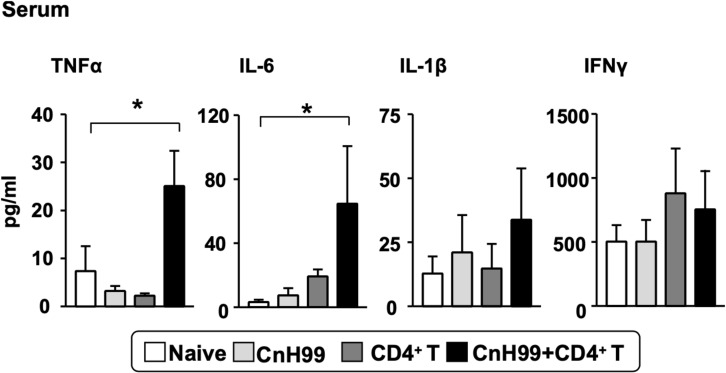
Serum proinflammatory cytokine levels. Serum cytokine levels of TNF-α, IL-6, IL-1β, and IFNγ were compared among 4 groups of mice with the protocol as indicated in Supplementary Figure 1A. Serum samples were obtained 4 weeks after *Cn* infection. C-IRIS received CD4^+^ T cell transfer 3 weeks after infection. *n = 5* mice per group for all experiments. The results are representatives from two independent experiments. One-way ANOVA and Tukey *post-hoc* test were used for statistical analyses; *p < 0.05.

In human C-IRIS, pro-inflammatory cytokine levels in serum increase dramatically after immune reconstitution (7, 31, 32). To assess systemic cytokine levels in our C-IRIS model, we examined four major pro-inflammatory cytokines in sera from C-IRIS mice with three control conditions (naïve, CnH99-infection alone, and CD4^+^ T cell transfer alone). C-IRIS mice showed significantly higher concentrations of TNFα and IL-6 in serum than control mice, while no notable differences were observed in IL-1β and IFNγ concentrations (Figure 2). The result indicates the systemic upregulation of specific inflammatory cytokines in C-IRIS.

### C-IRIS Enhances Lung Myeloid Cell Counts but Not Lung Fungal Burden

**FIGURE 3:**
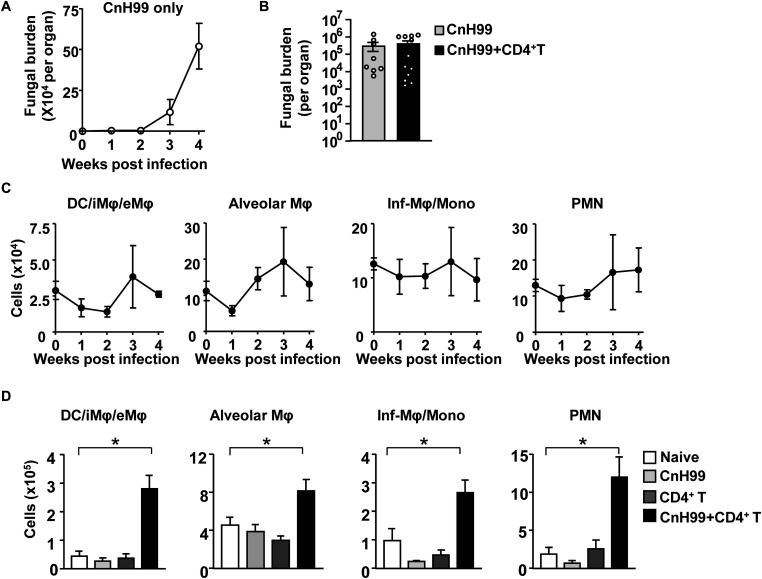
Fungal burdens and numbers of immune cells in the lung. **(A)** Lung fungal loads at indicated time-points (0, 1, 2, 3, and 4 weeks) post infection in a group with infection alone. **(B)** Comparison of lung fungal loads between mice with fungal infection alone (CnH99) and C-IRIS (CnH99 + CD4^+^ T cells) with the protocol indicated in Supplementary Figure 1A. One data-point denotes a result from one mouse. **(C,D)** Cell numbers of indicated myeloid cell types in the lung. Time-course data of a group with infection alone **(C)**. Comparison among 4 groups with the C-IRIS protocol indicated in Supplementary Figure 1A at 4 weeks after infection **(D)**. Flow cytometry was used for the analyses by determining DC, interstitial, and exudate macrophages (DC/iMΦ/eMΦ) as CD45^+^CD11c^+^F4/80^–^, alveolar macrophages (AM) as CD45^+^CD11c^+^F4/80^+^, inflammatory macrophage/monocyte as CD45^+^Ly6C^+^CD11b^+^, and neutrophils as CD45^+^Ly6G^*high*^CD11b^+^. *n = 5* mice per group for all experiments, except for **(B)**. The results are representatives from at least two independent experiments. One-way ANOVA and Tukey *post-hoc* test were used for statistical analyses; *p < 0.05.

We first examined the lung fungal burden in CnH99-infected *Rag1*^–^*^/^*^–^ mice without T cell transfer. Fungal burden steadily increased over 3 weeks after CnH99 infection and accelerated after week 3 (Figure 3A). Without T cell transfer, an increase in fungal burden was also found in the spleen but not as drastic as that observed in the lung (Supplementary Figure 1B). A previous report showed that a major risk factor in C-IRIS is high *Cn* burden in the lungs and the CNS (33). However, no difference in lung fungal loads was observed between groups with C-IRIS vs. infection alone at 4 weeks post infection (IRIS group had T cell transfer 7 days before the analysis) (Figure 3B). The result suggested that lung fungal loads do not explain the mortality of IRIS mice.

Next, we investigated numbers of major cell subtypes in the lung. The low infection inoculum in our protocol did not increase cell numbers of various myeloid cells without T cell transfer (Figure 3C). However, increased numbers of myeloid cells were observed in lungs of C-IRIS mice, compared to lungs of control groups (Figure 3D). These results suggest that C-IRIS does not increase lung fungal burdens, but enhances myeloid cell recruitment in the lung.

### Pathogenic Role of Th1 Cells in C-IRIS

**FIGURE 4:**
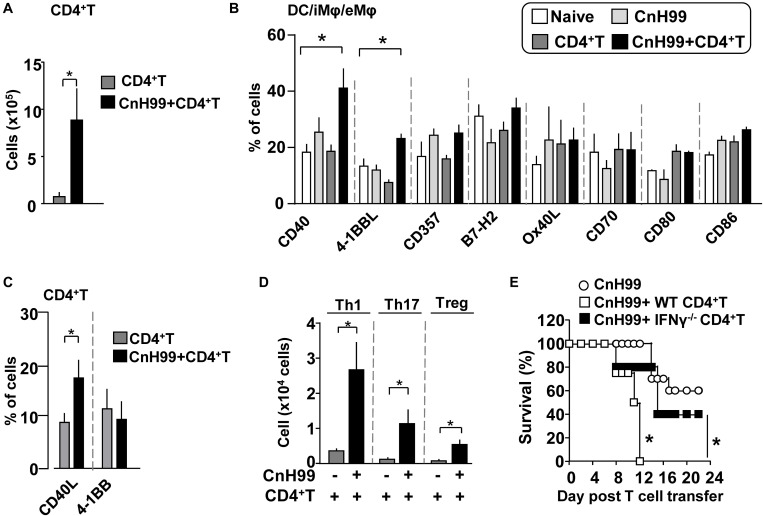
Evaluation of CD4^+^ T cells and APCs in C-IRIS. **(A)** Comparison of lung CD4^+^ T cell counts between groups with C-IRIS vs. T cell transfer alone. **(B)** Expression of co-stimulatory molecules on CD45^+^CD11c^+^F4/80^–^ cells. **(C,D)** Percentages of CD4^+^ T cells expressing CD40L or 4-1BB **(C)**. Numbers of Th1, Th17, and Treg cells in the lung **(D)**. **(E)** Survival C-IRIS-induced mice with *Ifng*^– /–^ CD4^+^ T cell or wild type CD4^+^ T cell. A control group with infection alone received vehicle (PBS with 2% FBS) injection at the same time with T cell transfer for test groups. Day 0 was set 3 weeks after infection. Except for **(E)**, evaluations were performed at 4 weeks post infection and/or 1 week after T cell transfer, and the treatment protocol of groups is indicated in Supplementary Figure 1A. *n = 5* mice per group for all experiments. The results are representatives from at least two independent experiments. Student’s *t*-test for **(A,C,D)**, one-way ANOVA and Tukey *post-hoc* test for **(B)**, and Log-rank analysis for **(E)** were used for statistical analyses; *p < 0.05.

C-IRIS mice had significantly more CD4^+^ T cells in the lungs than mice subjected to T cell transfer alone 7 days after T cell transfer (Figure 4A). Because the two groups received the same number of CD4^+^ T cells, the result suggested the significant increase of T cells in C-IRIS mice is involved in the pathology. To understand the potential mechanism, we evaluated the expression of T cell co-stimulatory molecule in CD45^+^CD11c^+^F4/80^–^ antigen-presenting cells (APCs), mainly dendritic cells (DCs). Among the eight co-stimulatory molecules examined, APCs in C-IRIS mice showed increased expressions of CD40 and 4-1BBL, although no significant difference was found in CD357, B7H2, Ox42L, CD70, CD80, and CD86 (Figure 4B). CD4^+^ T cells in C-IRIS mice also significantly increased the expression of CD40L, a CD40 ligand (Figure 4C). These results suggest an enhanced CD40-CD40L interaction between APCs and CD4^+^ T cells in the lung during C-IRIS. We also found that a majority of lung-infiltrated T helper cells in C-IRIS mice are Th1 cells (Figure 4D), similar to the lung in human C-IRIS (34, 35). To determine if our C-IRIS model is driven by Th1 cells, we transferred *Ifng*^–/–^ CD4^+^ T cells into CnH99 pre-infected *Rag1*^–^*^/^*^–^ mice and monitored their survival. C-IRIS-induced mice with *Ifng*^–/–^ CD4^+^ T cells indeed survived significantly longer than those with IFNγ-sufficient CD4^+^ T cells (Figure 4E). This result strongly suggests the involvement of Th1 cells in the pathogenicity of our C-IRIS model.

### C-IRIS Impacts the Brain More Than the Lung

**FIGURE 5:**
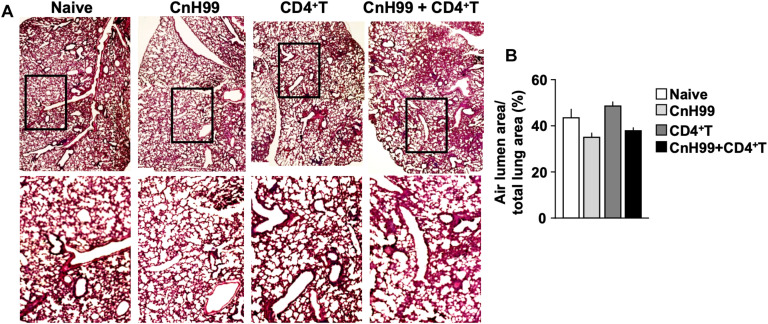
Evaluating lung pathology in C-IRIS mice. **(A)** Representative images of H&E-stained lungs collected from 4 groups with the protocol, indicated in Supplementary Figure 1A. **(B)** Quantitative analysis of areas of distal airspaces in lung sections, calculated from average values of at least 16 fields/mouse (more details are described in the section “Materials and Methods”); *n > 3* mice/group. The results are representatives from two independent experiments.

**FIGURE 6:**
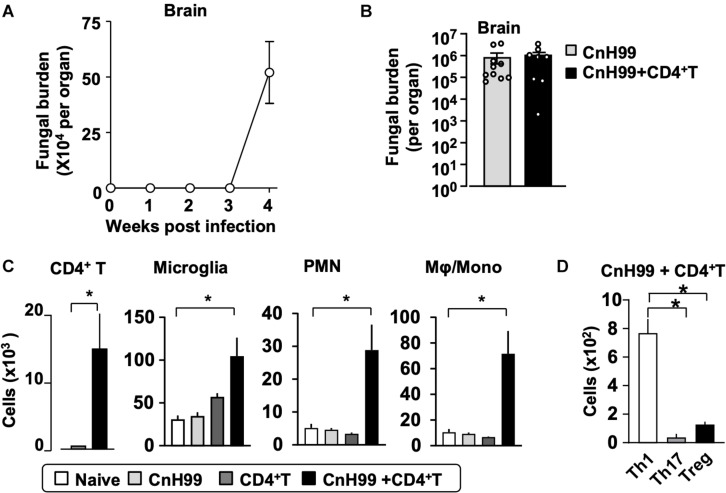
CNS fungal burden and immune cell infiltration. **(A)** Time-course of fungal loads in brains after CnH99 infection in a group with infection alone. **(B)** Comparison of brain fungal loads between mice with infection alone (CnH99) vs. C-IRIS (CnH99+CD4^+^ T cells). One data-point denotes a result from one mouse. **(C)** Numbers of indicated cell types in the brain of mice among four groups of mice. **(D)** Numbers of infiltrated Th1 (CD45^+^CD3^+^CD4^+^IFNγ^+^), Th17 (CD45^+^CD3^+^CD4^+^IL17^+^), Treg (CD45^+^CD3^+^CD4^+^Foxp3^+^) in the brain of C-IRIS mouse. Gating strategy for each cell type is shown in Supplementary Figure 3B. Except for **(A)**, evaluations were performed at 4 weeks post infection and/or 1 week after T cell transfer, and the treatment protocol of groups is indicated in Supplementary Figure 1A. *n = 5* mice/group for all experiments. The results are representatives from two independent experiments. Student’s *t*-test (**C**; CD4^+^ T cells) and one-way ANOVA and Tukey *post-hoc* test for (**C**; myeloid cells, **D**) were used for statistical analyses; *p < 0.05.

Fungal burden and leukocyte accumulation in the lung of C-IRIS mice led us to suspect lung damage as a cause for mortality. However, unexpectedly, C-IRIS mice did not exhibit significant histopathology in the lung (Figure 5A). Since it was difficult to tell histological differences by eye, we quantitatively evaluated the air sac areas in the histology images (Figure 5B). Nevertheless, C-IRIS mice still did not show abnormality and kept shapes of alveoli as well as other groups. Because our analyses suggest no abnormalities in the lung histology, we examined the brain. CnH99 is known to enter the brain via direct transmigration of the capillary endothelium or infected phagocytic cells (36). Before T cell transfer, no fungal burden was detected in the first 3 weeks but exponentially increased at week 4 (Figure 6A). However, we again found no difference in brain fungal loads between IRIS mice and CnH99-infected control mice without T cell transfer 7 days after C-IRIS mice received T cell transfer (Figure 6B). Because C-IRIS is often characterized by CNS inflammation, we enumerated leukocyte infiltrates and CNS-resident microglia in the C-IRIS brain. Although control groups (naïve, CnH99-infection alone, T cell transfer alone) did not show signs of inflammatory cell infiltration in the brain, C-IRIS mice showed clear infiltration of CD4^+^ T cells, macrophages/monocyte, and neutrophils, as well as increased numbers of microglia (Figure 6C). Additionally, a majority of brain-infiltrated T helper cells were Th1 cells in C-IRIS mice (Figure 6D).

**FIGURE 7:**
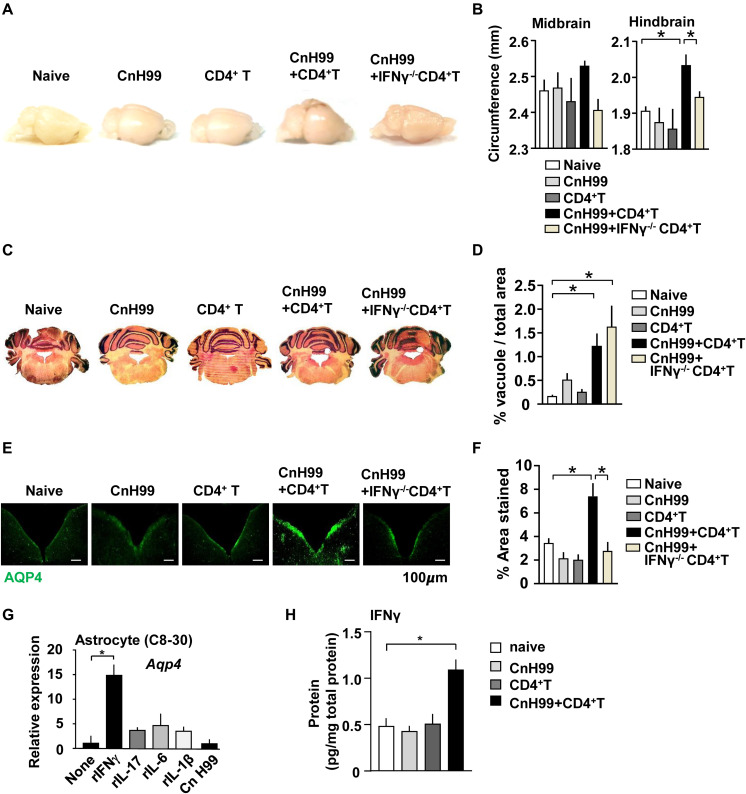
Analyses of the brain in C-IRIS. **(A,B)** Representative images of brains from 5 groups of mice **(A)**. Quantitative analysis of tissue circumference of midbrain and hindbrain from 5 groups of mice **(B)**. *n = 5–8* mice/group. **(C,D)** Representative images of H&E stained hindbrain sections from 5 groups **(C)** and quantitative analysis of brain void spaces **(D)**. *n = 5* mice/group. **(E,F)** Representative images of AQP4-stained brain stem sections **(E)** and quantitative analysis of stained area **(F)**. *n = 3–5* mice/group. **(G)** Expression levels of *Aqp4* mRNA in the C8-30 astrocytes, treated with indicated recombinant cytokines (10 ng/ml for all) or CnH99 (MOI of 10) for 5 h. *n = 5* biological data-points/group. **(H)** Hindbrain IFNγ protein levels, evaluated with protein lysates of the hindbrain by ELISA. *n = 5–9* mice/group. Mice were treated with the protocol, indicated in Supplementary Figure 1A in all the *in vivo* experiments. The results are representatives from two independent experiments. One-way ANOVA and Tukey *post-hoc* test were used in **B**, **D**, **F**, and **H**. The Student’s T test was used in **G**; *p < 0.05.

Gross phenotypical changes in the brain, such as edema was observed in C-IRIS mice 7 days after CD4^+^ T cell transfer but not in control cohorts (Figure 7A). The phenotype in C-IRIS mice was particularly significant in hindbrain, rather than midbrain (Figure 7B). Importantly, no increase of hindbrain circumference was observed when C-IRIS was induced with *Ifng*^–/–^ CD4^+^ T cells (Figures 7A,B). Tissue damage, identified with extra void spaces and vacuolization, were observed in hindbrain of C-IRIS mice 7 days after CD4^+^ T cell transfer (Figures 7C,D). Notably, tissue damage was also observed in C-IRIS-induced mice with *Ifng*^–/–^ CD4^+^ T cells, but to a lesser extent in mice with infection only (Figures 7C,D). We found that expression of AQP4, a main contributor to brain fluid homeostasis, was upregulated in the hindbrain of C-IRIS mice, while no AQP4 upregulation was observed in C-IRIS-induced mice with *Ifng*^–/–^ CD4^+^ T cells (Figures 7E,F). Previous studies demonstrated that transcription of *Aqp4* in astrocytes is upregulated by inflammatory cytokines (37, 38). Thus, we evaluated *Aqp4* gene expression by treating the C8-30 astrocyte cell line with recombinant (r) IFNγ, rIL-17, rIL-6, rIL-1β, or CnH99 for 5 h. *Aqp4* mRNA levels were significantly upregulated with IFNγ but not with other treatment conditions (Figure 7G). Although the behavior of astrocytes *in vivo* cannot be replicated by cell line experiments, the result at least suggested *Aqp4* expression in astrocytes can be induced by IFNγ. Therefore, we evaluated IFNγ levels in the hindbrain and found that C-IRIS mice showed elevated IFNγ protein levels in the hindbrain (Figure 7H). In sum, these results suggest that C-IRIS induces brain damage with edema characterized by enhanced IFNγ and *Aqp4* expression in the hindbrain.

## Discussion

Patients with C-IRIS present high CD4^+^ T cell numbers, particularly Th1 cells (6, 8), suggesting a crucial role for Th1 in C-IRIS development. A previous study of a C-IRIS mouse model used the less virulent *Cn* serotype D, 1841 strain and reported that C-IRIS is Th1-independent (39). The 1841 strain was originally isolated from human patients (40), as well as H99 (41). The 1841 C-IRIS model showed mild weight loss up to 6–7% with no mortality (39), possibly due to the low virulence of the strain. Therefore, the impact of Th1 cells may not have been clear in the hosts. In contrast, our model employs the highly virulent *Cn* serotype A, H99 strain. Unlike the 1841 C-IRIS model, our H99 C-IRIS model demonstrates rapid wasting and mortality, as well as Th1 responses, thus capturing the phenotype of dramatic deterioration and death as seen in some C-IRIS patients (4, 6, 7).

We asked if the pathogenicity of C-IRIS is attributed to possible systemic increase of proinflammatory cytokine levels. C-IRIS mice indeed showed enhanced cytokine levels but the levels are much lower than those seen in mice with LPS endotoxemia. For example, serum TNF-α levels in mice with LPS endotoxemia rise to 1,000–8,000 pg/ml (42), while C-IRIS mice had ∼25 pg/ml of blood TNF-α, as shown in Figure 2. Thus, the relatively mild increase of serum proinflammatory cytokines in C-IRIS mice does not appear to be significant enough to explain the acute death of the mice (Figure 1B).

Next, we speculated if the pathogenicity of C-IRIS results from lung injury triggered by lung inflammation and damage. First, numbers of lung infiltrated myeloid cells kept low without significant changes without T cell transfer. One possibility for this is the low inocula and immunogenicity of *Cn*, encapsulated for evasion from host detection (43). Our histology data also indicated minimal lung injury in C-IRIS mice, despite cellular infiltration including macrophages, neutrophils, and dendritic cells in the lung. In contrast to *Cn*, it is of note that *Mycobacterium avium* elicits strong innate immune responses and was used in a *Mycobacterium*-associated IRIS model (1, 44, 45), particularly exhibiting inflamed lung even before T cell reconstitution (46). In contrast, human C-IRIS is known to affect any organs, including the CNS (47). Thus, we were prompted to examine the brain, as neurological dysfunction has a serious impact on patient morbidity and mortality (48). Here, we demonstrated that the pathology of C-IRIS induced with CnH99 shows a sharp contrast to *Mycobacterium*-associated IRIS pathology, as C-IRIS does not result in severe lung inflammation despite its lethal phenotype.

In our C-IRIS model, distinct pathological changes were observed in the brain. It is possible that, in C-IRIS, more dramatic leukocyte infiltration and brain damages could ultimately hamper and cause failure of essential motor functions of the mice. In C-IRIS mice, we found increased numbers of CD4^+^ T cells, neutrophils, and microglia, as well as serious brain edema. Brain edema is caused by dysregulated water flux, which is controlled by aquaporins (AQPs). AQPs form a family of transmembrane channel proteins. Among 13 AQPs, only AQP4 is expressed in the brain (49, 50), where AQP4 is mainly expressed in astrocytes and plays a key role in regulating water flux in and out of the brain parenchyma (51, 52). In the CNS of *Cn-*infected mice, microglia may be activated in the vicinity of cryptococcal lesion, but the involvement of astrocytes were not studied for details (39). Overexpression of AQP4 induces brain edema (53), whereas AQP4 deletion reduces brain edema after acute water intoxication and ischemic stroke (54). We found heightened AQP4 expression in the brainstem of C-IRIS mice, as well as upregulation of *Aqp4* by IFNγ in an astrocyte cell line. We also demonstrated upregulation of *Aqp4* transcripts in an astrocyte cell line by IFNγ, as previously reported (55), as well as increased IFNγ protein levels in the brain of C-IRIS mice. Notably, no induction of AQP4 upregulation and brain edema were identified in mice received *Ifng*^–/–^ CD4^+^ T cells. Therefore, these results bring a new hypothesis that brain-infiltrated Th1 cells inducing AQP4 and resulting brain edema, which has a lethal impact. The possible involvement of Th1 cell-induced AQP4 in astrocytes needs to be defined *in vivo* in future studies.

Multiple datasets from this study suggested the pathogenic involvement of IFNγ, particularly in the brain. Nevertheless, C-IRIS mice have not increased serum IFNγ levels (Figure 2). The result at least indicates that the impact of IFNγ in C-IRIS is not exerted at the systemic level, but the increase of local IFNγ is detrimental enough. The involvement of pathogenic factors, other than IFNγ but somehow linked to IFNγ, is also possible.

To assess the extent of the C-IRIS animal model reflecting human C-IRIS, more thorough studies are awaited. For example, in this study, we did not evaluate serum antibody profiles, which was suggested to be candidate biomarkers of human C-IRIS (56). We do not fully rule out the involvement of pulmonary complications and a possible pre-condition of heightened innate immunity prior to reconstitution in C-IRIS, but our results suggest that the mortality of C-IRIS mice is mainly attributed to brain damage that may seriously deteriorate essential vital brain function. This study also suggests a blockade of IFNγ in the brain may be therapeutically effective in C-IRIS and nominates AQP4 as a novel target to ameliorate complications of intracranial pressure caused by C-IRIS. The interventions might be considered at the time of immune reconstitution, *e.g.*, antiretroviral therapy in HIV/AIDS. In conclusion, the mouse model of *Cn*−associated IRIS presented in this study provides a novel tool to unravel key mechanisms of pathogenesis.

## Data Availability Statement

All datasets generated for this study are included in the article/Supplementary Material. Raw data supporting the conclusions of this article will be made available by the authors without undue reservations.

## Ethics Statement

The animal study was reviewed and approved by IACUC of Duke University; IACUC of University of Illinois-Urbana Champaign.

## Author Contributions

MI, WB, and NA optimized the initial experimental conditions. MI, YK, NA, WB, and EK generated the data for this publication. All authors participated in experimental designs and data analysis. YK, MI, and MS wrote the manuscript. WB and NA edited the manuscript. MI and MS equally contributed to design overall direction of the project.

## Conflict of Interest

The authors declare that the research was conducted in the absence of any commercial or financial relationships that could be construed as a potential conflict of interest.

## Footnotes

1 https://imagej.net/Thresholding
