# Supplementary material for: Th1-Dependent Cryptococcus-Associated Immune Reconstitution Inflammatory Syndrome Model With Brain Damage
Source: Front Immunol. 2020 Sep 29;11:529219. doi: 10.3389/fimmu.2020.529219 (PMC7550401; doi:10.3389/fimmu.2020.529219)
Supplement: Supplementary file 1 [file Data_Sheet_1.PDF]

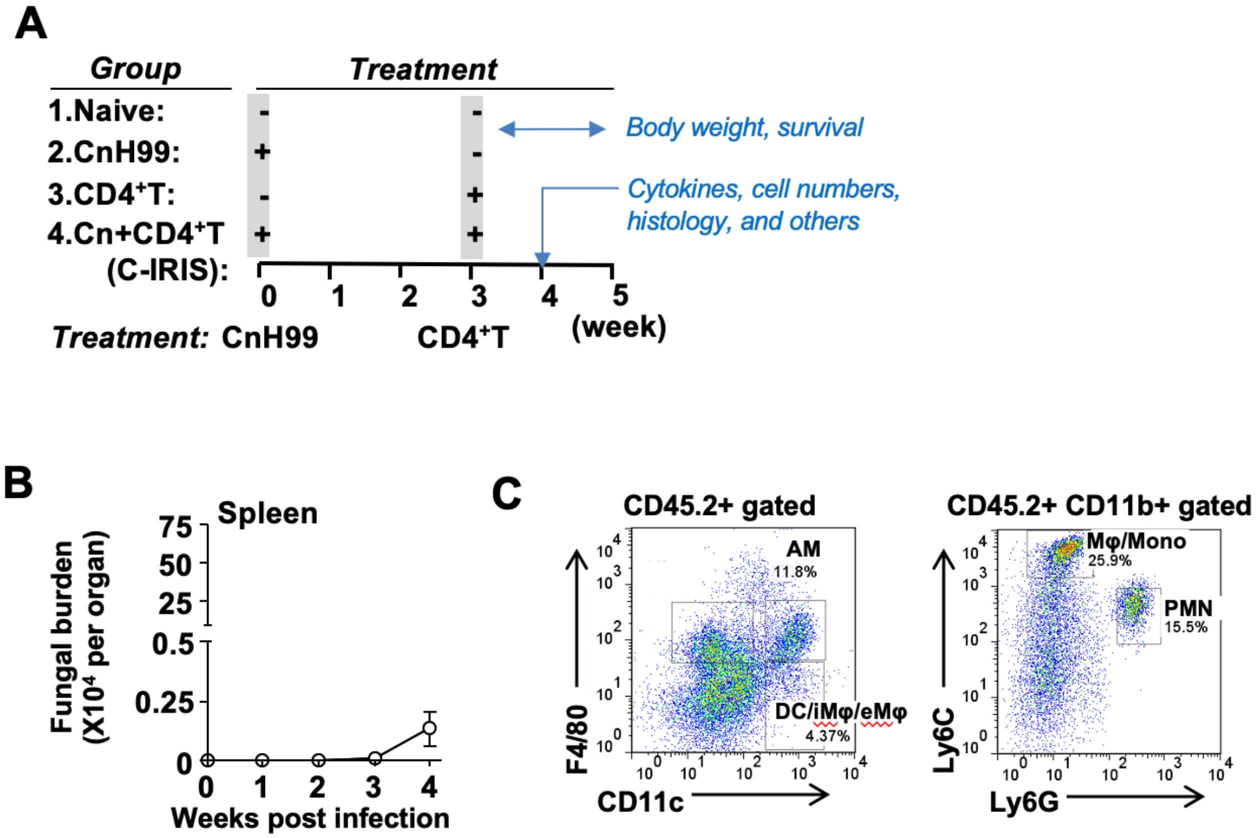

Supplementary Figure 1. Analyzing C-IRIS mice and control mice. (A) Schematic diagram of our C-IRIS induction protocol, which constitutes CnH99 infection in 16-20 weeks-old mice, followed by CD4+ T cell transfer 3 weeks post-infection, unless otherwise noted. There are three control groups: 1) naïve mice that receive neither Cn infection nor CD4+ T cell transfer, 2) "CnH99" mice that receive Cn infection only, 3) "CD4+ T" mice that receive CD4+T cells only. Except for body weight and survival analysis, mice were analyzed 4 weeks after infection, i.e., 7 days after T cell transfer for relevant groups. **(B)** CnH99 fungal loads in the spleen at indicated time-points after CnH99 infection in a group with infection alone. **(C)** Gating strategy for myeloid cells in the lung.

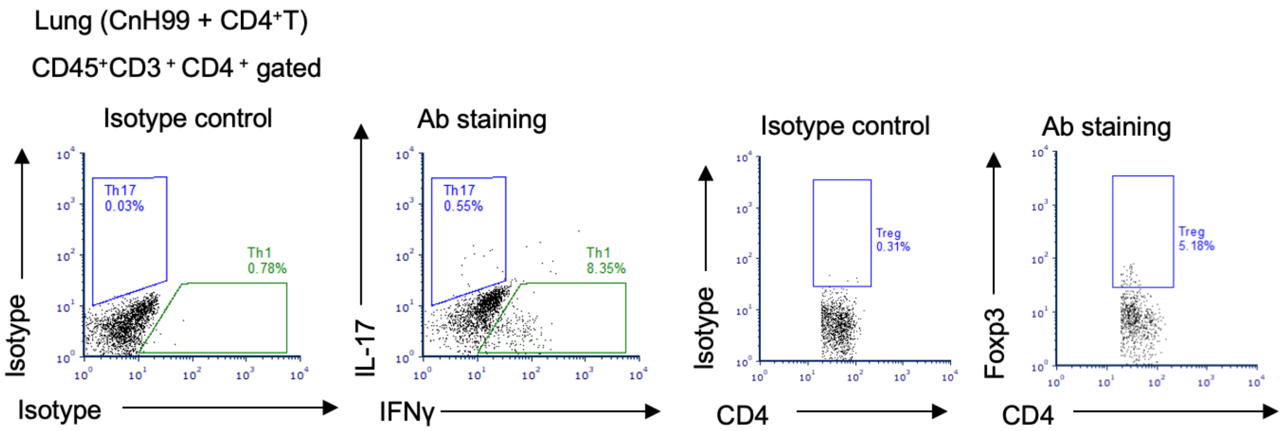

Supplementary Figure 2. Gating strategy for Th1, Th17, and Treg in the lung.

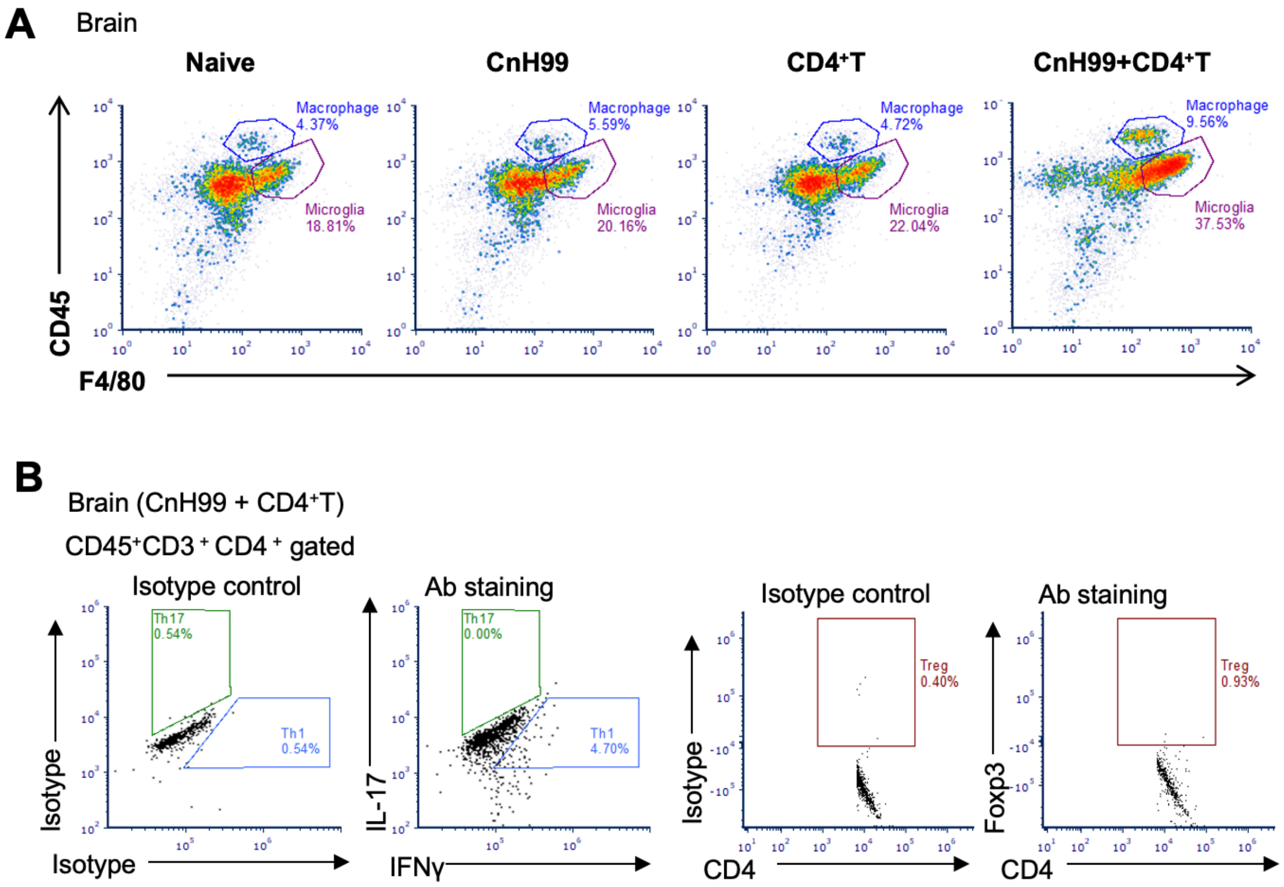

Supplementary Figure 3. Gating strategy for brains cells. (A) Gating strategy for microglia and macrophages in the brain. **(B)** Gating strategy for Th1, Th17, and Treg cells in the brain.

Supplementary Table 1 List of antibodies

Vender	Catalog #	Product
Biolegend	103210	PE/Cy5 anti-mouse/human CD45R/B220 Antibody
Biolegend	117322	Pacific Blue™ anti-mouse CD11c Antibody
Biolegend	127608	PE anti-mouse Ly-6G Antibody
Biolegend	101210	PE/Cy5 anti-mouse/human CD11b Antibody
Fisher	MF48021	F4/80 Monoclonal Antibody (BM8), Alexa Fluor 647
Biolegend	128006	FITC anti-mouse Ly-6C Antibody
Biolegend	103154	APC/Fire™ 750 anti-mouse CD45 Antibody
Biolegend	123108	FITC anti-mouse F4/80
Biolegend	104706	FITC anti-mouse CD80 Antibody
Biolegend	100406	FITC anti-mouse CD4 Antibody
Biolegend	107105	PE anti-mouse 4-1BB Ligand (CD137L) Antibody
Biolegend	108811	APC anti-mouse CD252 (OX40L) Antibody
Biolegend	100236	APC anti-mouse CD3 antibody
Biolegend	124621	PE/Cy7 anti-mouse CD40 Antibody
Biolegend	126317	PE/Cy7 anti-mouse CD357 (GITR) Antibody
Biolegend	107405	PE anti-mouse CD275 (B7-H2, B7-RP1, ICOS Ligand) Antibody
Biolegend	105030	APC/Cy7 anti-mouse CD86 Antibody
Biolegend	106511	PE/Cy7 anti-mouse CD154 Antibody
Biolegend	106105	PE anti-mouse CD137 Antibody
Biolegend	100310	PE/Cy5 anti-mouse CD3ε Antibody
Biolegend	100422	PE/Cy7 anti-mouse CD4 Antibody
Biolegend	506904	PE anti-mouse IL-17A Antibody
Biolegend	123108	FITC anti-mouse F4/80
Biolegend	126408	Alexa Fluor® 647 anti-mouse FOXP3 Antibody
Biolegend	101212	APC anti-mouse CD11b antibody
Biolegend	117306	FITC anti-mouse CD11c Antibody
Fisher	NBP1-87679	Rabbit Polyclonal Aquaporin-4 Antibody
Fisher	A11008	Goat anti-rabbit Alexa Fluor® 488 secondary antibody

Supplementary Table 2. List of primers

Primers name	Sequence 5' to 3'
Aqp4-Fw	GCT TAG ATC TGG CTT TCA AAG G
Aqp4-Rev	AAT GTC CAC ACT TAC CCC AC
Actb-Fw	TGT TAC CAA CTG GGA CGA CA
Actb-Rev	CTG GGT CAT CTT TTC ACG GT
